# Prognostic Value of *SPOCD1* in Esophageal Squamous Cell Carcinoma: A Comprehensive Study Based on Bioinformatics and Validation

**DOI:** 10.3389/fgene.2022.872026

**Published:** 2022-05-11

**Authors:** Zhizhong Lin, Lin Chen, Tingting Wu, Yiping Zhang, Xinyi Huang, Yuanmei Chen, Junqiang Chen, Yuanji Xu

**Affiliations:** ^1^ Department of Radiation Oncology, Fujian Medical University Cancer Hospital, Fujian Cancer Hospital, Fuzhou, China; ^2^ The School of Nusing, Fujian Medical University, Fuzhou, China; ^3^ Shengli Clinical Medical College of Fujian Medical University, Fuzhou, China; ^4^ Department of Thoracic Surgery, Fujian Medical University Cancer Hospital, Fujian Cancer Hospital, Fuzhou, China

**Keywords:** SPOCD1, esophageal squamous cell carcinoma, novel biomarkers, prognosis, immune infiltration

## Abstract

In the study, we aimed to explore and analyze the potential function of SPOC Domain Containing 1 (SPOCD1) in esophageal squamous cell carcinoma (ESCC). We performed a comprehensive analysis of gene expression of SPOCD1 and its corresponding clinicopathological features in ESCC. In particular, the correlation between SPOCD1 and ESCC was evaluated using a wide range of analysis tools and databases, including TCGA, GTEx, GenePattern, CellMiner, GDSC, and STRING datasets. Different bioinformatics analyses, including differential expression analysis, mutation analysis, drug sensitivity analysis, function analysis, pathway analysis, co-expression network analysis, immune cell infiltration analysis, and survival analysis, were carried out to comprehensively explore the potential molecular mechanisms and functional effects of SPOCD1 on the initiation and progression of ESCC. The expression of SPOCD1 was upregulated in ESCC tissues compared to those in normal tissues. In the high SPOCD1 expression group, we found apparent mutations in TP53, TTN, and MUC16 genes, which were 92, 36, and 18%, respectively. GO and KEGG enrichment analysis of SPOCD1 and its co-expressed genes demonstrated that it may serve as an ESCC oncogene by regulating the genes expression in the essential functions and pathways of tumorigenesis, such as glycosaminoglycan binding, Cytokine-cytokine receptor interaction, and Ras signaling pathway. Besides, the immune cell infiltration results revealed that SPOCD1 expression was positively correlated with Macrophages M0 and Mast cells activated cells, and negatively correlated with plasma cells and T cells follicular helper cell infiltration. Finally, ESCC patients with high expression of SPOCD1 indicated poor overall survival. qRT-PCR demonstrated that the SPOCD1 expression in ESCC tissues was significantly higher than adjacent tissues (*p* < 0.001). Our study indicated that SPOCD1 was increased in ESCC tissues. The current data support the oncogenic role of SPOCD1 in the occurrence and development of ESCC. Most importantly, SPOCD1 might be an independent prognostic factor for ESCC patients.

## Introduction

Esophageal cancer (EC) is the sixth most common cause of cancer death and the seventh most common malignant tumor globally, with a survival rate of less than 20% (Torre et al., 2015; Bray et al., 2018). EC is mainly divided into two pathological types: esophageal squamous cell carcinoma (ESCC) and esophageal adenocarcinoma (EA). ESCC is the predominant type in Asian countries (Abnet et al., 2018). Despite recent progress in surgery, radiotherapy, chemotherapy, and immunotherapy, the 5-years survival rate of ESCC patients remains dismal (Pennathur et al., 2013; Wang et al., 2018; Xu et al., 2019). The outcome of ESCC remains extremely poor, which is mainly due to its insensitive to chemotherapy and rapid progress. Due to the lack of reliable biomarkers, most patients have experienced the advanced stage at the time of diagnosis. Distant metastasis usually occurs at this stage, resulting in a poor outcome. In addition, patients with advanced ESCC often suffer from severe pain that makes it difficult to be treated. Therefore, it is in dire need to find promising diagnostic biomarkers and novel therapeutic targets of ESCC, which will help reveal the molecular mechanisms of ESCC tumorigenesis and metastasis.

SPOC Domain Containing 1 (SPOCD1), also known as PPP1R146, is a protein coding gene. An important paralog of this gene is Death Inducer-Obliterator 1 (DIDO1). It encodes a protein that belongs to the transcription factor S-II (TFIIS) family of transcription factors. Alternate splicing results in multiple transcript variants. It is widely expressed in many tissues and has been reported in various tumors. Most notably, some studies have explored the biological function of SPOCD1 in human cancer, such as gastric cancer, clear cell renal cell carcinoma, ovarian cancer, osteosarcoma, and glioma (Zhu et al., 2017; Liang et al., 2018; Liu et al., 2018; Sakaguchi et al., 2018; Liu et al., 2020). In addition, some studies have demonstrated that SPOCD1 is an essential executor of piRNA-directed *de novo* DNA methylation in recent years (Zoch et al., 2020). These studies revealed the underlying value of SPOCD1 in tumors and may provide a new direction for further understanding the specific role and molecular mechanism of SPOCD1. Nonetheless, there is no study of SPOCD1 in ESCC at present. In addition, SPOCD1 is considered to be an attractive therapeutic and prognostic target for cancer, and inhibition of SPOCD1 may be a feasible cancer treatment strategy. Consequently, we selected SPOCD1 as the research object and explored the potential function and mechanism of SPOCD1 through various kinds of analyses in ESCC.

So far, the analysis on the SPOCD1 role in ESCC remains largely unknown. In the present study, we performed a comprehensive analysis of the gene expression of SPOCD1 and its corresponding clinicopathological features in ESCC. In particular, the correlation between SPOCD1 and ESCC was evaluated using a wide range of analysis tools and databases, including TCGA, GTEx, GenePattern, CellMiner, GDSC, and STRING datasets. Different bioinformatics analyses, including differential expression analysis, single nucleotide polymorphism (SNP)/copy number variation (CNV) analysis, drug sensitivity analysis, function analysis, pathway analysis, co-expression network analysis, immune cell infiltration analysis, and survival analysis, were carried out to comprehensively explored the potential molecular mechanisms and functional effects of SPOCD1 on the initiation and development of ESCC. Furthermore, we explored the expression of SPOCD1 in ESCC tissues, and the expression was validated by quantitative real-time polymerase chain reaction (qRT-PCR) in 21 ESCC patients. Together, the study provides evidence for the clinical and functional significance of SPOCD1, as well as its potential as a novel strategy for cancer treatment, providing momentous evidence for exploring the role of SPOCD1 in ESCC.

## Methods

### Sample Collection and Pretreatment

Esophageal squamous cell carcinoma (ESCC) tissues (n = 6) and adjacent normal esophageal tissues (n = 6) were retrieved from surgical patients in Fujian Cancer Hospital from July 1st to 17 July 2020. All patients were not treated before surgery. The clinical data for RNA sequencing of patients with esophageal squamous cell carcinoma in our institution was shown in Supplementary Table S1. The specimens after surgical resection were immediately placed in liquid nitrogen and then transferred to the refrigerator at −80°C for preservation. All patients involved in the study received written informed consent for biological research. The research scheme (including specimen collection) was reviewed and approved by the Biomedical Ethics Committee of the Fujian Cancer Hospital (batch number: K2021-027-01). All procedures were in line with the guidelines of the Helsinki Declaration of the World Medical Association. The clinicopathological staging and classification of the patients were in accordance with the criteria of the American Joint Committee on Cancer (AJCC).

**FIGURE 1:**
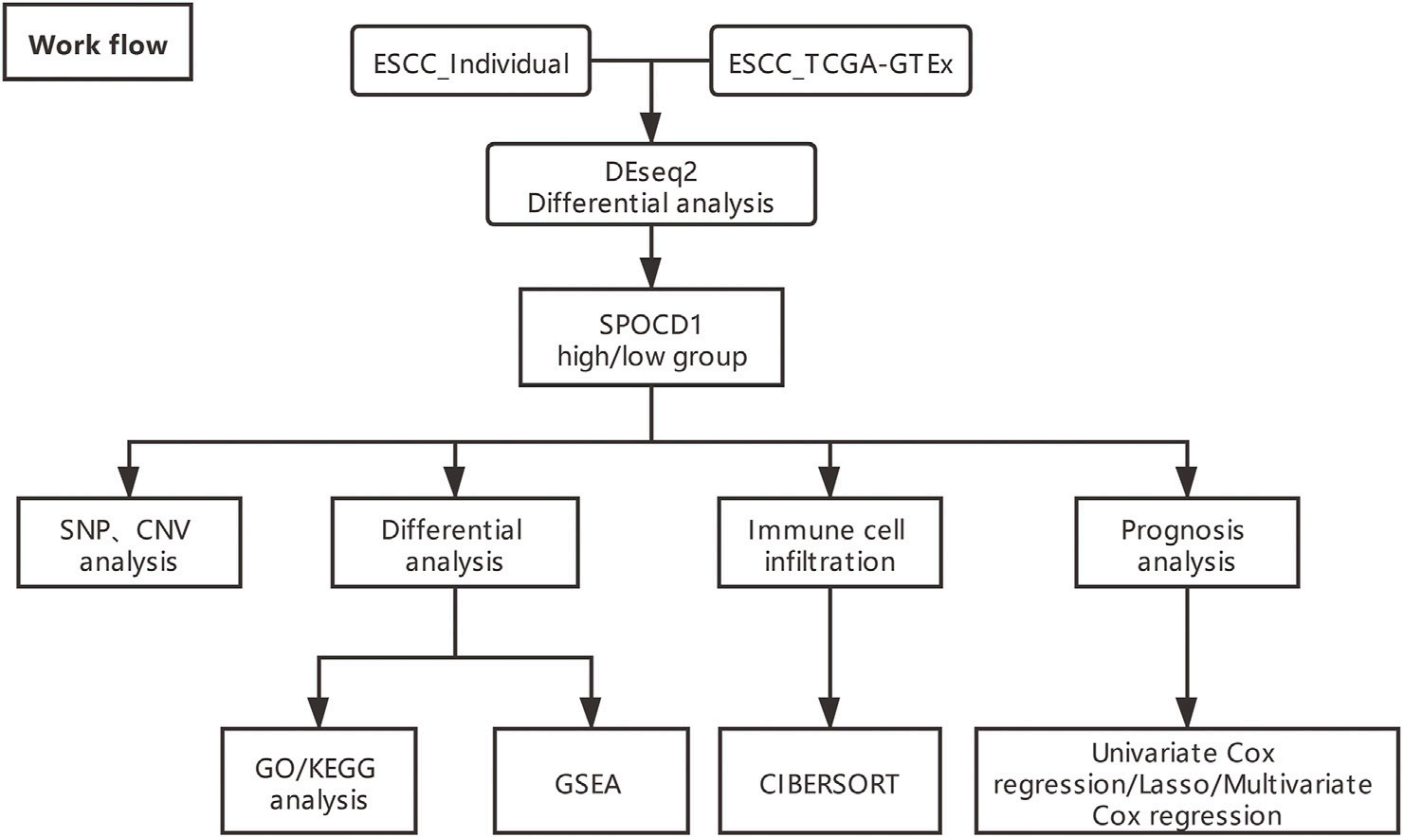
The overall analysis flow chart (ESCC_Individual is self-test data). ESCC, esophageal squamous cell carcinoma; TCGA, The Cancer Genome Atlas; SPOCD1, SPOC Domain Containing 1; SNP, single nucleotide polymorphism; CNV, copy number variation; GO, Gene Ontology; KEGG, Kyoto Encyclopedia of Genes and Genomes; GSEA, gene set enrichment analysis.

In addition, the count data, SNP data, CNV, and matched clinical data (n = 78) of ESCC RNA-seq were downloaded from The Cancer Genome Atlas (TCGA) database (https://portal.gdc.cancer.gov/projects/) by GDC software. Due to the deficiency of normal control sample (n = 1) in ESCC in TCGA database, the RNA-seq count data of esophageal normal control samples (n = 663) combined with TCGA combined with GTEx and ESCC samples (n = 78) were obtained by UCSC Xena (https://xenabrowser.net/datapages/) (The data was corrected in batches by combat package). The analysis process was shown below (Figure 1).

### RNA Sequencing

The above six pairs of esophageal tissues were used for the sequencing of RNA. The total RNA was extracted by RNA extraction kit (Qiagen). The samples were sequenced on Illumina HiSeq and produced an average of 42.38 million reads. We next used the TopHat2 (v2.1.1) to map them to the human reference genome (GRCh38/hg38). In addition, the splicing sequence of RNA were identified by the CIRC explorer program (v2.2.3) together with the fusion connection gained from the TopHat2. RNA quantification, quality evaluation, library construction, and sequencing were carried out by Shanghai Life genes Biotechnology Co., Ltd. Raw data in fastq format was processed through perl scripts. We also use HTSeq v0.6.1 (https://htseq.readthedocs.io/en/master/) calculation to get the read count and TPM of each gene.

### Screening of Differential Genes Between Normal and Tumor Samples

The COUNT value of difference analysis of RNA sequencing data of TCGA, GTEx and our medical center were standardized before difference analysis. First of all, the principal component analysis (PCA) analysis of the sequencing data of our center and the TCGA combined with the GTEx data set was carried out through the FactoMine R package (Lê et al., 2008). Then Deseq2 R package (Love et al., 2014) was used to screen the differentially expressed genes between tumor and normal samples in our center and TCGA combined with GTEx data set. The differential genes met the requirements of *p*-value < 0.05 and | log2 Fold Change | > 1. The differential genes obtained from the two data sets were intersected, and the interested gene of SPOCD1 was selected for follow-up analysis. Finally, we employed ggpubr R package (ggpubr, 2017) to draw the expression box diagram of SPOCD1 in the two data sets.

### Analysis of SPOCD1 Expression and Mutation Data

According to the expression of SPOCD1, the tumor samples of our center and TCGA-ESCC were divided into high SPOCD1 expression group and low SPOCD1 expression group, and the differential genes in the high expression group and low expression group were screened by the Deseq2 package. The differential genes satisfied the requirements of *p*-value < 0.05 and | log2 Fold Change | > 1. Besides, the heat map and volcano map were drawn by heatmap R package (Kolde, 2015) and ggplot2 R package (Ginestet, 2011), respectively, to show the overall expression and differential expression of SPOCD1-related genes.

The expression of SPOCD1 in various tissues of the human body was sorted out by GTEx database (https://gtexportal.org/home/), and visualized by ggplot2 R package. The somatic mutation data of TCGA-ESCC patients were extracted by maftools R package (Mayakonda and Koeffler, 2018). The somatic mutation data of patients with high expression of SPOCD1 were collected and analyzed to excavate the high frequency mutation genes of patients with high expression of SPOCD1. Subsequently, we identified statistically significant amplification and deletion in the high SPOCD1 expression group and the low SPOCD1 expression group by using genomic identification of essential targets in cancer (GISTIC) 2.0 (https://cloud.genepattern.org/gp/pages/index.jsf).

### Related Drug Analysis of SPOCD1

The data of mRNA expression profile and drug activity of SPOCD1 gene were downloaded from CellMiner database (https://discover.nci.nih.gov/cellminer/). CellMiner is a web-based tool that contains genomic and pharmacological information for researchers to use transcripts and drug response data from NCI-60 cell lines compiled by the US. National Cancer Institute (Reinhold et al., 2012). The transcriptional expression levels of 22,379 genes, 360 microRNA, and 20,503 compounds in drug responses are available on the CellMiner website. We calculated the correlation between SPOCD1 gene expression and compound sensitivity by Pearson correlation analysis.

In addition, we used the genomics of drug sensitivity in cancer (GDSC) database (www.cancerrxgene.org/) (Yang et al., 2013) and pRRophetic algorithm (Paul et al., 2014) to construct a ridge regression model based on GDSC cell line expression profile and TCGA gene expression profile, which contributes to predict the IC50 values of high and low SPOCD1 expression groups for common anticancer drugs.

### Functional Enrichment Analysis

ClusterProfiler R package (Yu et al., 2012) was used to analyze the enrichment of differential genes by Gene Ontology (GO) and Kyoto Encyclopedia of Genes and Genomes (KEGG), respectively. Adj. *p*-value < 0.05 was considered to be statistically significant. Pathview R package (Luo and Cory, 2013) was used to visualize the pathways of KEGG enrichment. The gene expression matrix was analyzed by the gene set enrichment analysis (GSEA) using clusterProfiler R package, and “c2. cp.kegg.v7.0. symbols.gmt” was selected as the reference gene set. False discovery rate (FDR) < 0.25.

### Analysis of PPI Interaction Network and miRNA-Hub Gene Regulatory Network

The STRING protein-protein interaction database (https://www.string-db.org/) was performed to analyze the interaction between genes. After the results were derived, the hub genes were further screened by the CytoHubba plug-in in Cytoscape (Chin et al., 2014). In addition, Networkanalyst was used to regulate and analyze the Hub gene-miRNA, and the miRNA-target gene was predicted according to the “minimum number of network connections”. Finally, the results were derived from Networkanalyst, and the miRNA-hub gene regulatory network was drawn by Cytoscape software.

### Analysis of Immune Cell Infiltration and Correlation Analysis

CIBERSORT (http://CIBERSORT.stanford.edu/) and LM22 characteristic gene matrices were used to predict the proportion of 22 immune cells in all samples of the data set. We used CIBERSORT R package (Newman et al., 2015) to evaluate the abundance of 22 kinds of immune cells in the TCGA-ESCC data set and calculate the correlation between 22 kinds of immune cells. Then, by integrating the information of SPOCD1 expression, the Pearson correlation between the expression of SPOCD1 and immune cell infiltration was further calculated.

### Prognostic Analysis and Model Construction of SPOCD1

Firstly, we analyze the survival of SPOCD1 in the TCGA-ESCC dataset through the survival R package (Therneau, 2012). In addition, to obtain SPOCD1-related prognostic genes, we conducted a preliminary screening by univariate Cox regression with a *p*-value < 0.1. Then glmnet R package (Friedman et al., 2010) was used to carry out lasso regression to further remove overfitted variables. Finally, independent prognostic factors were determined by multivariate cox regression analysis, and the results were visualized by a risk heat map. Among them, the formula for calculating the prognostic risk score was as follows: risk score = expression value of gene 1 *β1 + expression value of gene 2 *β2 +… + expression value of gene n * βn, where β is the regression coefficient obtained in the process of calculation. According to the results of multivariate cox analysis, independent prognostic factors were screened according to *p* < 0.05, and risk heat map and scatter map were drawn.

### qRT-PCR Validation for the Expression of SPOCD1

Twenty-one ESCC tissues and adjacent tissues from ESCC patients were obtained from July 2020 to June 2021 in Fujian Cancer Hospital. The clinical data for qRT-PCR of patients with esophageal squamous cell carcinoma in our institution was shown in Supplementary Table S2. qRT-PCR was applied to verify the expression of the target SPOCD1 using twenty-one pairs of ECA and adjacent normoal esophageal tissues. The primers of SPOCD1 were listed in Table 1, purchased from BioSune (Shanghai, China). An RTⅢ All-in-One Mix with dsDNase (Monad Biotech Co., Ltd, Shanghai, China) was used to synthesize cDNA from 1 µg of total RNA. The qRT-PCR analyses were conducted on the StepOnePlus Real-Time PCR System (Applied Biosystems, Thermo Fisher Scientific Co., Ltd, US) by the Hieff ®qPCR SYBR^®^ Green Master Mix, High Rox (Yeasen, Biotechnology Co., Ltd, Shanghai, China). The reaction was: 95°C for 10 min, then 41 cycles of 95°C for 15 s and 60°C for 1 min, last 95°C for 15 s. The reference gene was GAPDH, and the relative levels of gene expression were calculated by the 2^−ΔΔCt^ method.

**TABLE 1:** The primers of SPOCD1 and GAPDH.

Primers	Forward primers(5′ to 3′)	Reverse primers**(**5′ to 3′**)**
qPCR-SPOCD1	CAT​GGA​CCA​GAC​ACT​GAC​CC	CTC​CCA​GTC​CTT​GCA​GAT​GT
qPCR-GAPDH	GGA​GCG​AGA​TCC​CTC​CAA​AAT	GGC​TGT​TGT​CAT​ACT​TCT​CAT​GG

### Statistical Analysis

All tests were two-sided, and a *p*-value less than 0.05 was considered to indicate a statistically significant difference. The statistical analysis was performed with R software (version 4.0.2).

## Results

### Screening of Differential Genes Between Normal and Tumor Samples

**FIGURE 2:**
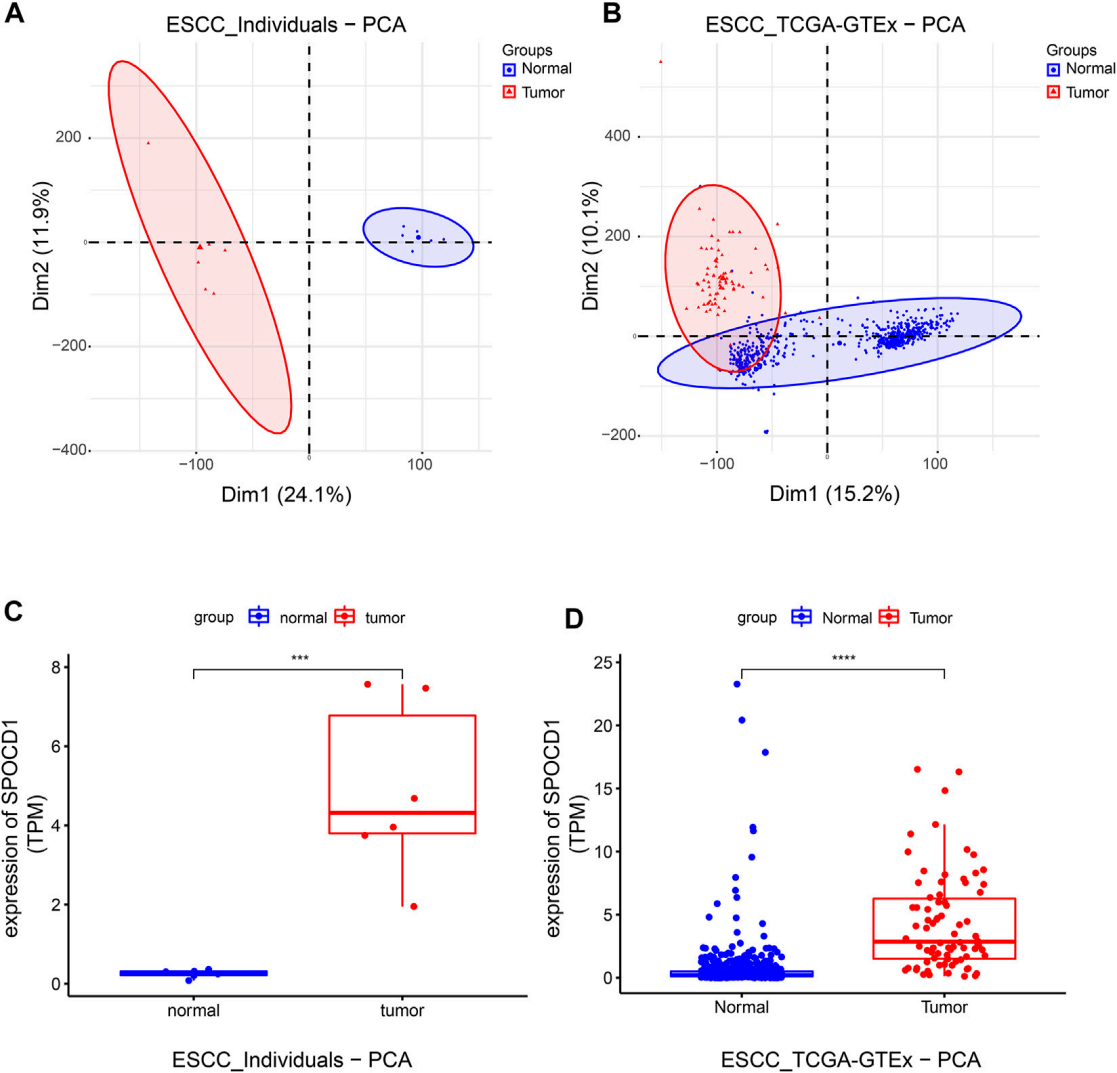
The grouping of tumor and normal samples and the detection of SPOCD1 expression. **(A)** The results of PCA analysis of the sequencing data of our center samples; **(B)** The results of PCA analysis of the sequencing data of TCGA combined with GTEx dataset samples; **(C)** The sequencing data of our center showed that the expression of SPOCD1 in esophageal squamous cell carcinoma was significantly higher than that in the normal group; **(D)** The sequencing data of TCGA combined with GTEx data set showed that the expression of SPOCD1 in esophageal squamous cell carcinoma was significantly higher than that in the normal group. ***p < 0.001, ****p < 0.0001. SPOCD1, SPOC Domain Containing 1; ESCC, esophageal squamous cell carcinoma; TCGA, The Cancer Genome Atlas; PCA, principal component analysis.

We sorted out the sequencing data of our center and the TCGA combined with the GTEx data set. The results of PCA analysis showed that the normal samples and tumor samples were completely separated in our center, and most of the normal samples and tumor samples were separated in the data set of TCGA and GTEx. The two data sets indicated a good state of sample separation (Figures 2A,B). We then used the Deseq2 R package to analyze the differences in count data. Finally, 4,435 differential genes were found in the sequencing data of our center, and 4,126 differential genes were found in the TCGA combined with the GTEx data set. After screening, the SPOCD1 gene was discovered to be differentially expressed in both data sets.

Consequently, we compared the difference of SPOCD1 between tumor samples and normal samples in the two data sets after selecting SPOCD1 as the target gene. It was revealed that the expression of SPOCD1 in tumors was significantly higher than that in normal tissues (Figures 2C,D). The clinical data of patients with esophageal squamous cell carcinoma in TCGA was listed in Table 2.

**TABLE 2:** Clinical data of patients with esophageal squamous cell carcinoma in TCGA.

Characteristic	Levels	Overall
N		95
Gender, n (%)	Female	15(15.79%)
	Male	80(84.21%)
Age, n (%)	≤60	56(58.95%)
	>60	39(41.05%)
Pathologic stage, n (%)	Stage I	14(14.74%)
	Stage II	59(62.11%)
	Stage III	14(14.74%)
	Stage IV	8(8.42%)
OS event, n (%)	Alive	77(81.05%)
	Dead	18(18.95%)

### Related Gene Analysis of SPOCD1

**FIGURE 3:**
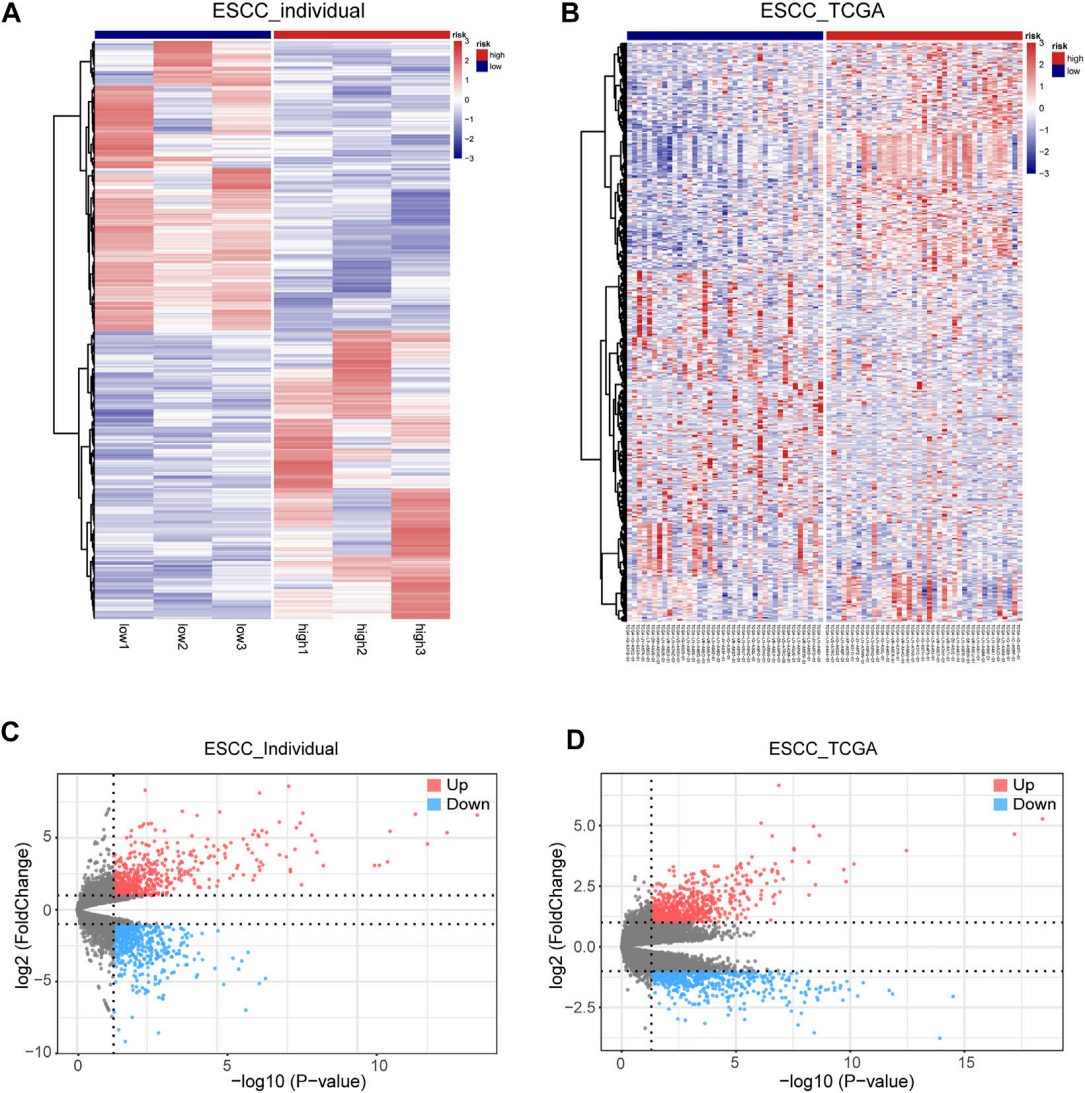
The condition of differential expression of high SPOCD1 expression group and low SPOCD1 expression group. **(A,B)** The heat map of differentially expressed genes in high and low SPOCD1 expression was divided into two groups according to the median value of SPOCD1. Red represents upregulated differential genes, and blue represents downregulated differential genes; **(C,D)** The volcano map of differential genes showed that red represents upregulated differential genes, blue represents downregulated differential genes, and gray represents non-differential genes. SPOCD1, SPOC Domain Containing 1; ESCC, esophageal squamous cell carcinoma; TCGA, The Cancer Genome Atlas.

Based on the expression value of SPOCD1, we divided the tumor samples from our center data and TCGA data set into high SPOCD1 expression group and low SPOCD1 expression group according to the median value. It was shown that a total of 1,335 differential genes were found in our center tumor data set and 1,295 differential genes were found in the TCGA tumor data set. The volcanic map depicted the expression level of differential genes, and the overall difference was displayed by the heat map (Figures 3A–D).

### Correlation Analysis of SPOCD1 Expression and Mutation

**FIGURE 4:**
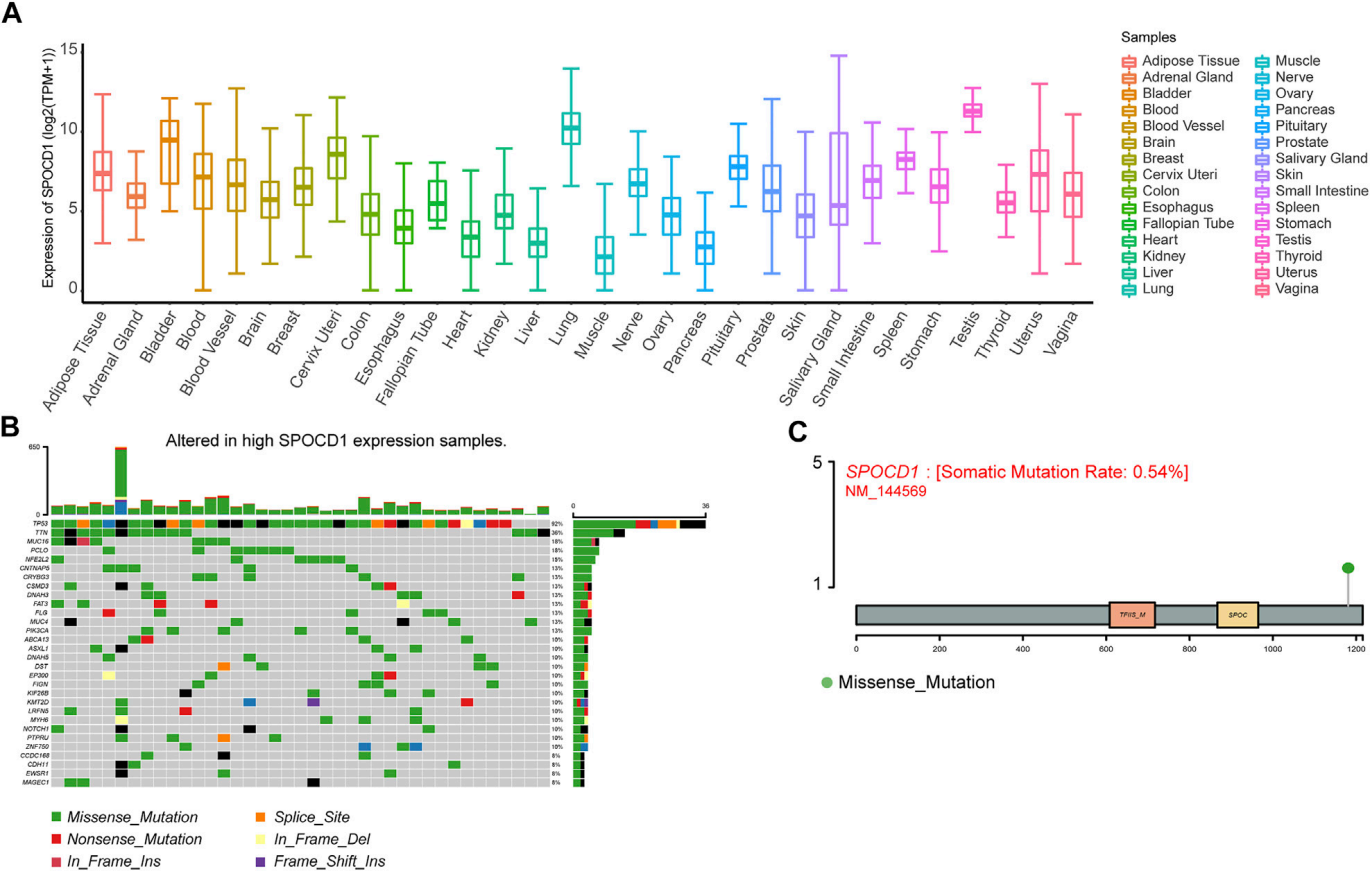
Correlation analysis of SPOCD1 expression and mutation. **(A)** The expression of SPOCD1 in human tissues; **(B)** Mutant panorama of high SPOCD1 expression group; **(C)** Map of SPOCD1 mutation site information. SPOCD1, SPOC Domain Containing 1; CNV, copy number variation.

To explore the expression characteristics of SPOCD1 in human tissues, we extracted the expression data of SPOCD1 from the GTEx database. The results showed that it was highly expressed in testis tissue and lung tissue (Figure 4A). As for ECSS, the tumor patients in TCGA-ESCC were divided into two groups: high expression of SPOCD1 group and low expression of SPOCD1 group. In the high expression group, we found apparent mutations in tumor protein P53 (TP53), Titin (TTN), and mucin 16 (MUC16) genes, which were 92, 36, and 18%, respectively (Figure 4B). Based on the mutation site of SPOCD1, we mapped the mutation information of SPOCD1 (Figure 4C). In addition, by collating the CNV information of TCGA-ESCC, we calculated the changes of CNV in the groups with high expression of SPOCD1 and low expression of SPOCD1 by the GISTIC 2.0 algorithm. As shown in Supplementary Figure S1, there was no significant change in CNV in the groups between high and low expression of SPOCD1.

### Drug Sensitivity Analysis and Drug Prediction

**FIGURE 5:**
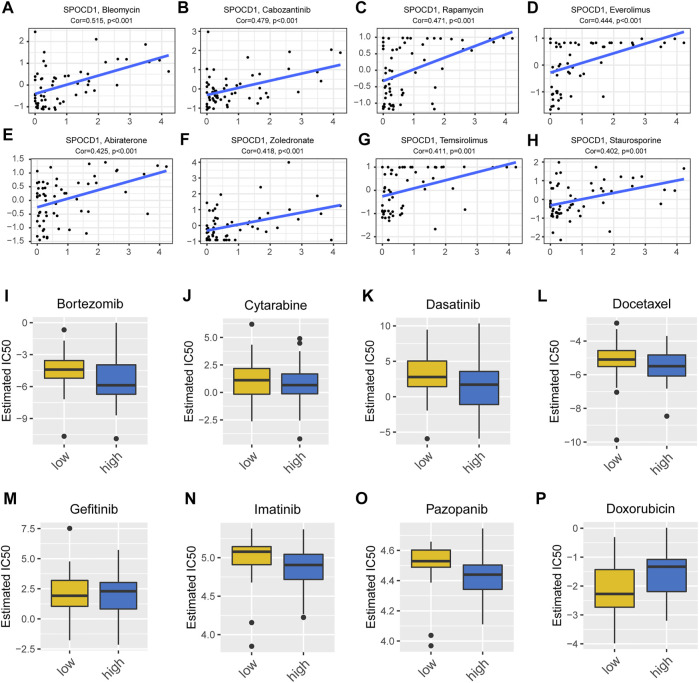
Drug sensitivity analysis and drug prediction. **(A–H)** The top eight drugs with the most significant correlation with SPOCD1 in CellMiner database; **(I–P)** The differences in IC50 values for drugs prediction with high and low SPOCD1 expression from the GDSC database. SPOCD1, SPOC Domain Containing 1; GDSC, genomics of drug sensitivity in cancer.

In order to evaluate the potential effect of SPOCD1 on drug response, we analyzed the Pearson correlation between the expression of the SPOCD1 gene in the NCI-60 cell line and the activity of antineoplastic drugs retrieved from CellMiner database. According to the order of correlation, we selected the first eight drugs most related to AURKA (Aurora Kinase A). AURKA is a protein coding gene. The protein encoded by this gene is a cell cycle-regulated kinase that appears to be involved in microtubule formation and/or stabilization at the spindle pole during chromosome segregation. The encoded protein is found at the centrosome in interphase cells and at the spindle poles in mitosis. This gene may play a role in tumor development and progression. A processed pseudogene of this gene has been found on chromosome 1, and an unprocessed pseudogene has been found on chromosome 10. As shown in Figure 5, there was a positive correlation between SPOCD1 and Bieomycin, Cabozantinib, Rapamycin, Everolimus, Abiraterone, Zoledronoate, Temsirolimus, and Staurosporine (Figures 5A–H). In addition, we analyzed the IC50 value of drugs in high and low expression groups of SPOCD1 through the GDSC database. No significant difference was presented in common antineoplastic drugs such as cisplatin (*p* > 0.05) (Supplementary Figure S2), but there were significant differences in Bortezomib and Doxorubicin (*p* < 0.05) (Figures 5I–P).

### Functional Enrichment Analysis

**FIGURE 6:**
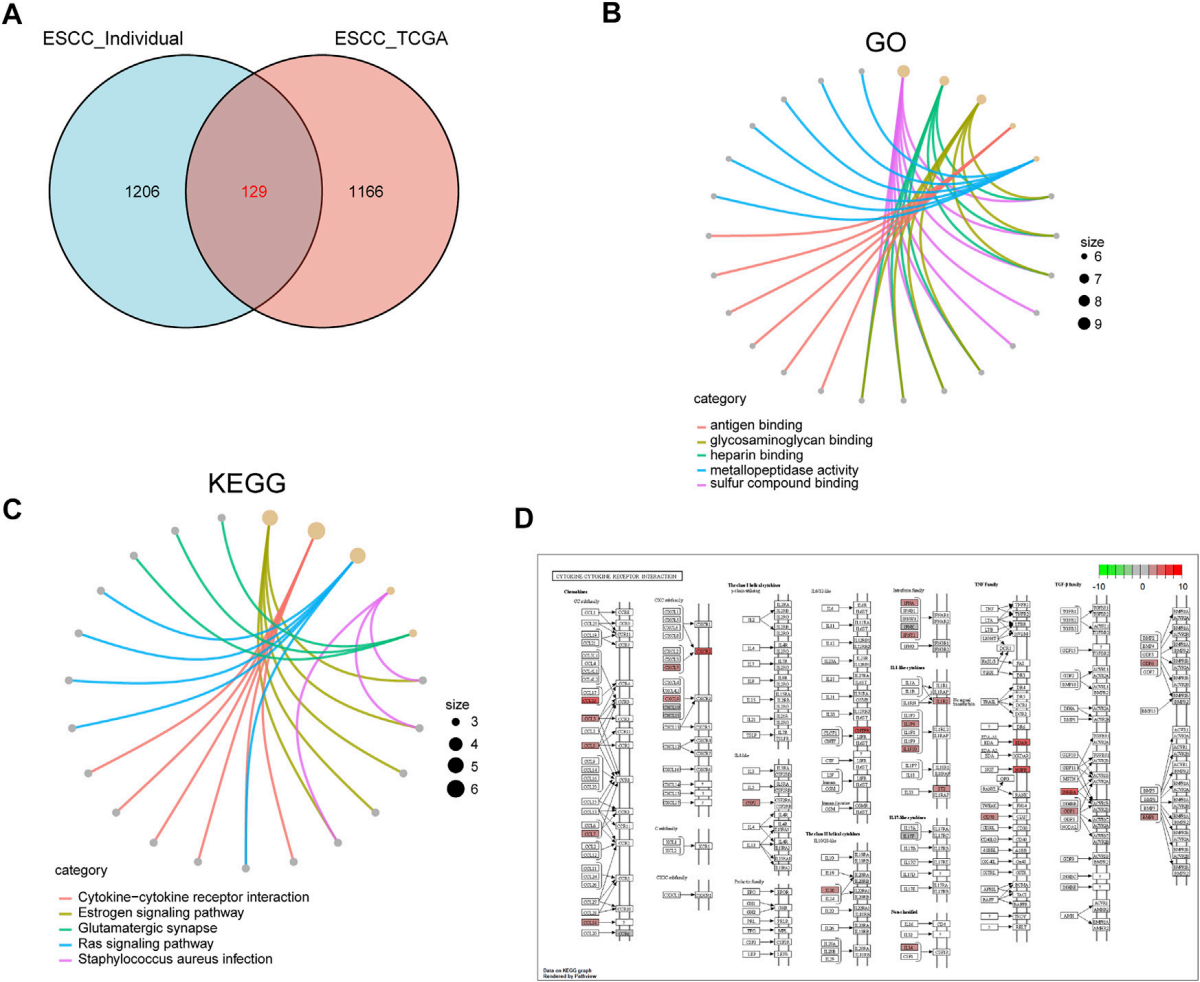
GO and KEGG analysis of differentially expressed genes in high and low SPOCD1 expression groups. **(A)** The differentially expressed genes of high and low SPOCD1 expression group in the sequencing data of our center and TCGA data set were intersected; **(B)** GO biological function enrichment analysis, the size of the dot represents the proportion of gene enrichment in this function; **(C)** KEGG pathway enrichment analysis, the annotation is the same as before; **(D)** Cytokine-cytokine receptor interaction, the pathway of significant enrichment of KEGG; the red represents the degree of gene enrichment, the redder the color, the more obvious the upregulation of the gene in the pathway; the greener the color, the more obvious the downregulation of the gene in the pathway. GO, Gene Ontology; KEGG, Kyoto Encyclopedia of Genes and Genomes; SPOCD1, SPOC Domain Containing 1; TCGA, The Cancer Genome Atlas.

**FIGURE 7:**
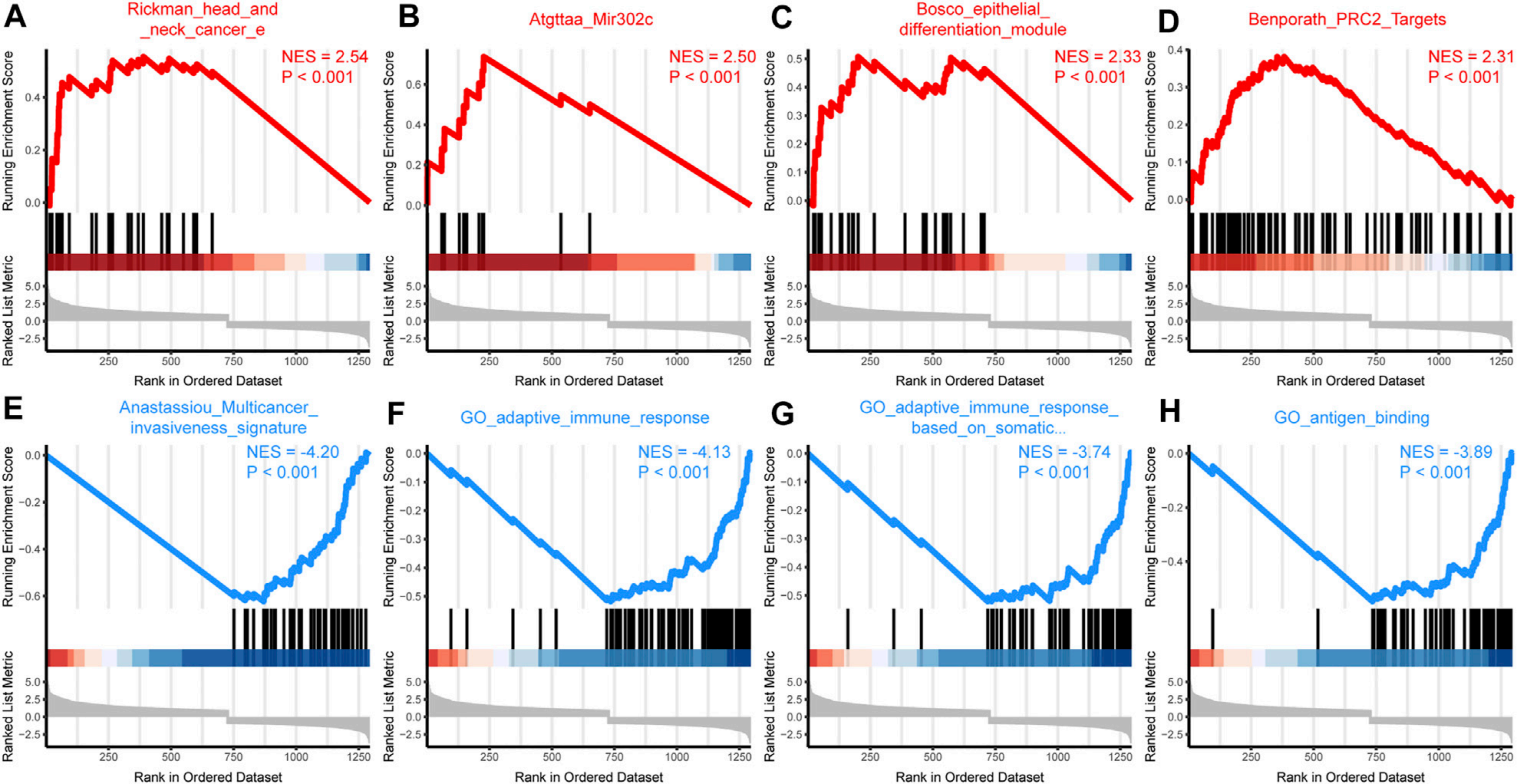
GSEA enrichment analysis. **(A–D)** According to the NES value, the differentially expressed genes were enriched in the first four pathways in the high SPOCD1 expression group. The NES value represents the normalized enrichment fraction, and the higher the NES value is, the more genes are enriched in this pathway. The *p*-value reflects the credibility of the enrichment results; **(E–H)** According to the NES value, the differentially expressed genes were enriched in the first four pathways in the low SPOCD1 expression group. The NES value represents the normalized enrichment fraction, and the higher the NES value is, the more genes are enriched in this pathway. The *p*-value reflects the credibility of the enrichment results. GSEA, gene set enrichment analysis; NES, normalized enrichment score; SPOCD1, SPOC Domain Containing 1.

To further explore the function of SPOCD1, we intersected the differential genes obtained from the high and low SPOCD1 expression groups in the two data sets. Finally, 129 common differential genes were identified (Figure 6A). GO analysis showed that these differential genes were mainly associated with sulfur compound binding, heparin binding, and glycosaminoglycan binding (Figure 6B). The results of the KEGG analysis were shown in Figure 6C. The pathways of these differential genes were enriched mainly including the Estrogen signaling pathway, Cytokine-cytokine receptor interaction, Ras signaling pathway, and *Staphylococcus aureus* infection. At the same time, we further demonstrated the pathway of hsa04060: Cytokine-cytokine receptor interaction, which enriches most genes (Figure 6D). Lastly, the pathway of GSEA enrichment mainly referred to Rickman head and neck cancer, Atgttaa miR302c, Bosco epithelial differentiation module, Benporath PRC2 Targets, Anastassiou Multicancer invasiveness signature, GO adaptive immune response, GO adaptive immune response Based on somatic, and GO antigen binding (Figures 7A–H). NES stands for normalized Enrichment score. Because the ES is calculated according to whether the gene in the analyzed dataset appears in a functional gene set, but the number of gene contained in each functional gene set is different, and the correlation between different functional gene set and data is also different. Therefore, it is necessary to standardize the ES to compare the enrichment degree of dataset in different functional gene set. That is, the NES = ES of a function gene set/the average ES of all random combinations of the dataset. NES is the main statistic. NES >0, this pathway is enriched in high expression group. NES <0, this pathway is enriched in low expression group. The GO, KEGG, and GSEA enrichment analysis results of differential genes were shown in Tables 3–5, respectively.

**TABLE 3:** Results of GO enrichment analysis of differential genes.

ID	Description	p.adjust	geneID	Count
GO:1,901,681	sulfur compound binding	0.01	COL5A3/FGFR1/SMOC1/GSTM3/GSTM2/PLA2G2D/FGF9/CCL8/CXCL11	9
GO:0008201	heparin binding	0.01	COL5A3/FGFR1/SMOC1/PLA2G2D/FGF9/CCL8/CXCL11	7
GO:0005539	glycosaminoglycan binding	0.03	COL5A3/FGFR1/SMOC1/PLA2G2D/FGF9/CCL8/CXCL11	7
GO:0003823	antigen binding	0.03	IGHV3-72/IGLV3-21/IGHV3-66/LILRB4/KLRC2/IGHV4-34	6
GO:0008237	metallopeptidase activity	0.04	MMP20/CPA6/VASH2/MMP12/MMP9/MMP8	6

**TABLE 4:** Results of KEGG enrichment analysis of differential genes.

ID	Description	*p*-value	Count
hsa04915	Estrogen signaling pathway	0.00139	5
hsa04060	Cytokine-cytokine receptor interaction	0.00827	6
hsa04014	Ras signaling pathway	0.01258	5
hsa05150	Staphylococcus aureus infection	0.02001	3
hsa04724	Glutamatergic synapse	0.03124	3
hsa04930	Type II diabetes mellitus	0.03128	2
hsa04010	MAPK signaling pathway	0.03143	5
hsa04973	Carbohydrate digestion and absorption	0.03254	2
hsa00520	Amino sugar and nucleotide sugar metabolism	0.03383	2
hsa04015	Rap1 signaling pathway	0.03741	4
hsa05207	Chemical carcinogenesis - receptor activation	0.03853	4
hsa00480	Glutathione metabolism	0.04626	2

**TABLE 5:** Results of GSEA enrichment analysis.

Description	setSize	NES	p.adjust	Rank
SCHUETZ_BREAST_CANCER_DUCTAL_INVASIVE_UP	69	−4.221612585	**2.86E-08**	525
ANASTASSIOU_MULTICANCER_INVASIVENESS_SIGNATURE	42	−4.197893077	2.86E-08	426
GO_ADAPTIVE_IMMUNE_RESPONSE	77	−4.126203037	2.86E-08	561
POOLA_INVASIVE_BREAST_CANCER_UP	58	−4.04773592	2.86E-08	540
BENPORATH_PRC2_TARGETS	76	2.30528993	0.000990457	382
BOSCO_EPITHELIAL_DIFFERENTIATION_MODULE	27	2.325602524	0.00687682	201
ATGTTAA_MIR302C	11	2.501002207	0.000703345	227
RICKMAN_HEAD_AND_NECK_CANCER_E	27	2.54007954	0.000990457	389

### Protein-Protein Interaction Network and miRNA-Target Gene Regulatory Network

**FIGURE 8:**
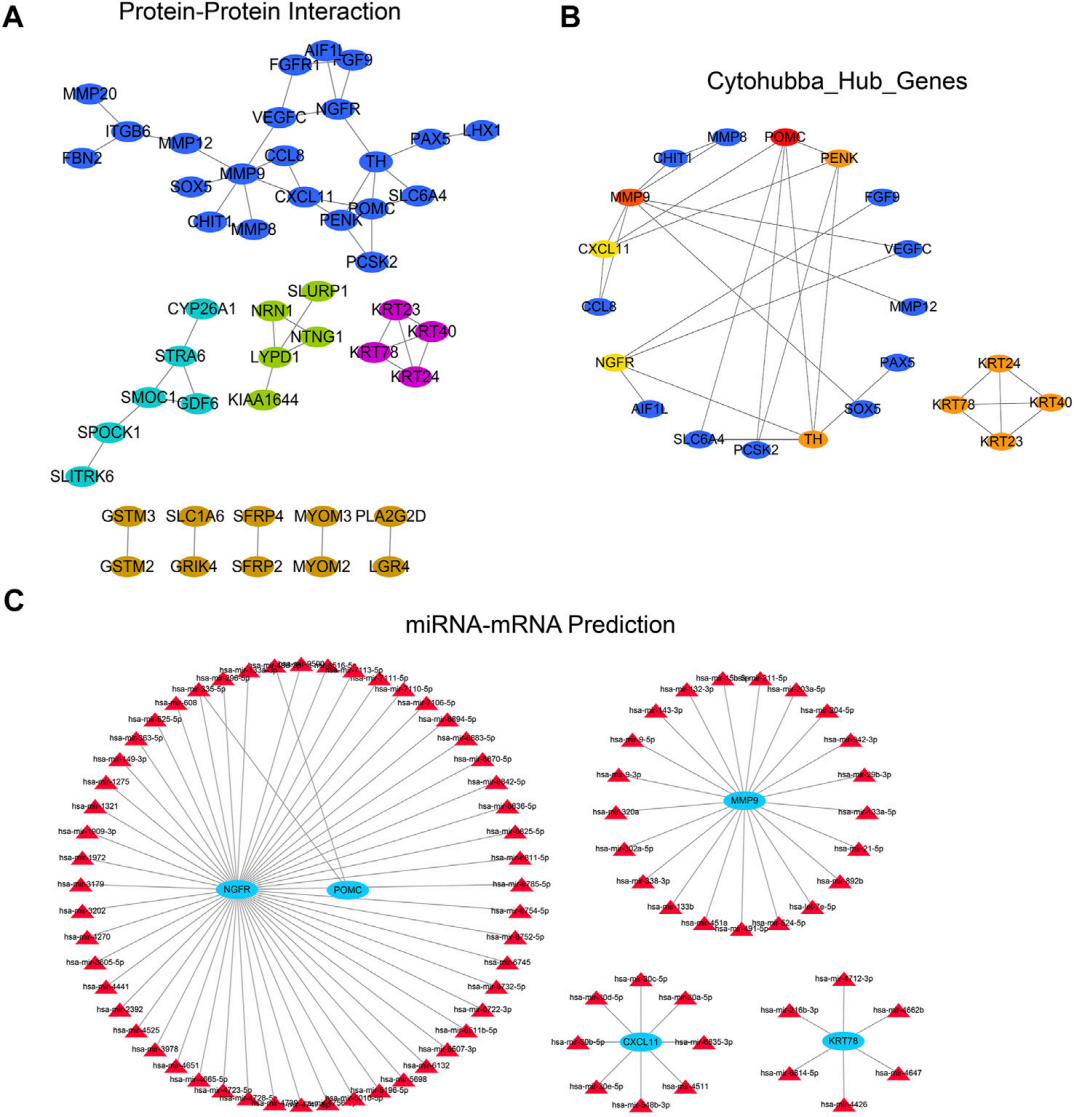
PPI and miRNA regulatory network analysis of differential genes. **(A)** PPI regulatory network established by differential genes; **(B)** Two subnetworks and 10 hub genes were further screened by Cytohubba plug-in; **(C)** According to the miRNA-hub gene regulatory network predicted by the Networkanalyst database to regulate hub genes. The Networkanalyst database predicted the miRNA-hub gene regulatory network of regulatory hub genes.PPI, protein-protein interaction.

We constructed a PPI network based on the String database to reveal the potential relationship between differential genes. When the minimum interaction score was 0.4, only 47 of the 129 differential genes interacted with other gene pairs (Figure 8A). The PPI network consists of 47 characteristic genes and 51 edges, and the average node degree was 0.93. Then we further identified the most relevant genes in the PPI network through the Cytohubba plug-in. We also used the cytohuabba plug-in in cytoscape to screen for differential genes. After screening, Cytohubba identified 10 genes that can be regarded as hub genes: POMC, PENK, TH, NGFR, CXCL11, MMP9, KRT24, KRT40, KRT23, and KRT78 (Figure 8B). Then, we predicted the 10 potential miRNA regulating hub genes through the Networkanalyst database, and the screening condition was “Trim current network to minimum connected network” (minimum link network). Finally, four miRNA regulatory subnetworks are obtained, including 4 nodes, 89 edges, and 87 seeds (Figure 8C).

### Immune Cell Infiltration and Correlation Analysis

**FIGURE 9:**
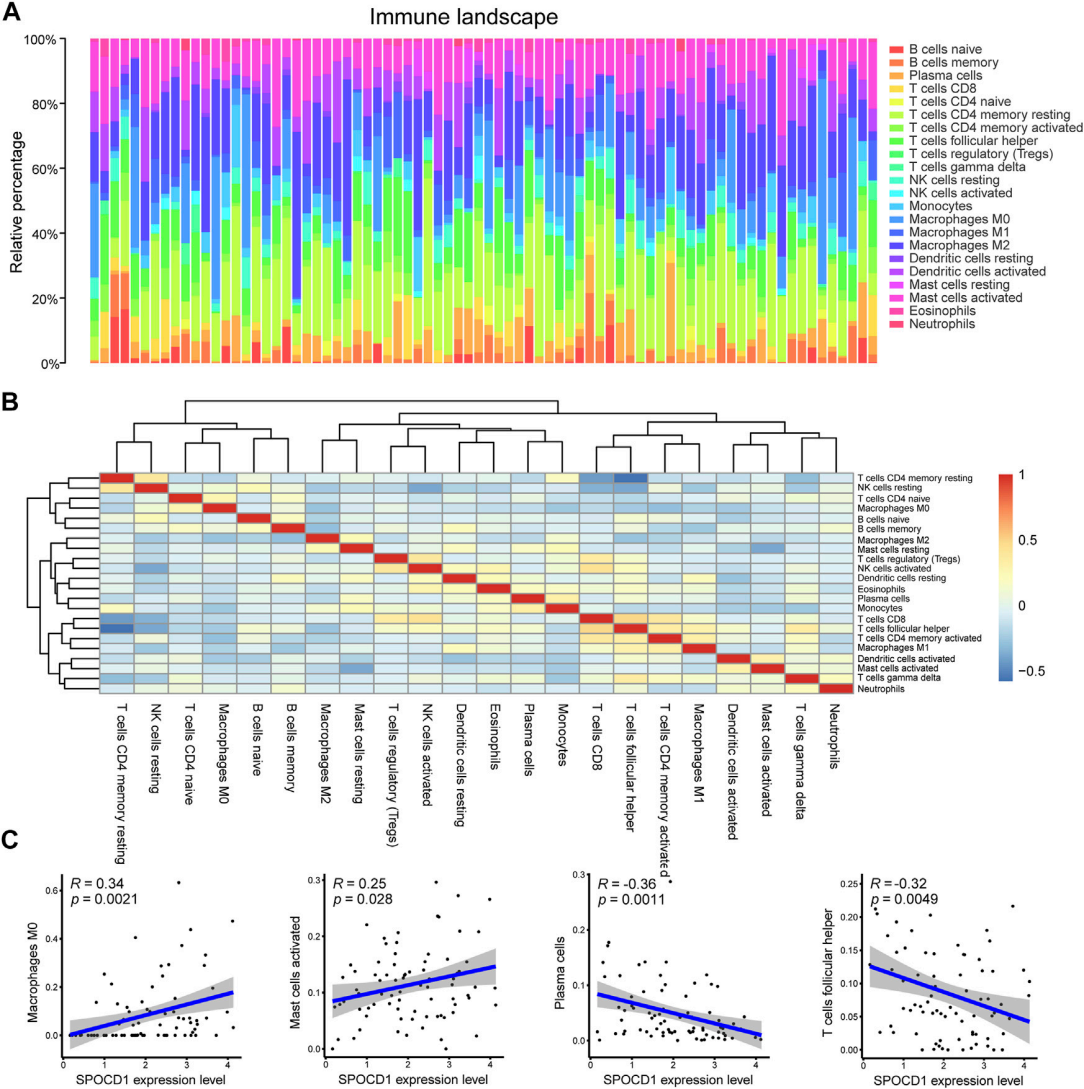
Evaluation and correlation analysis of immune cell infiltration. **(A)** Panoramagram of 22 kinds of immune cell infiltration in TCGA-ESCC tumor samples; **(B)** Correlation heat map of 22 kinds of immune cell score; **(C)** Correlation between SPOCD1 gene expression and immune cell infiltration. TCGA, The Cancer Genome Atlas; ESCC, esophageal squamous cell carcinoma; SPOCD1, SPOC Domain Containing 1.

To analyze the relationship between the expression of SPOCD1 and immune cell infiltration, we calculated the proportion of immune cell infiltration in the tumor microenvironment by the CIBERSORT algorithm. Figure 9A and Figure 9B demonstrated the panorama of immune cell infiltration in the tumor microenvironment of ESCC and the correlation of immune cell score, respectively. After integrating SPOCD1 gene expression and immune cell infiltration score, we comprehensively analyzed the infiltrating immune cells, which were significantly correlated with SPOCD1 expression. The results showed that SPOCD1 expression was positively correlated with Macrophages M0 and Mast cells activated cells, and negatively correlated with plasma cells and T cells follicular helper cell infiltration (Figure 9C).

### Prognosis Analysis of SPOCD1

**FIGURE 10:**
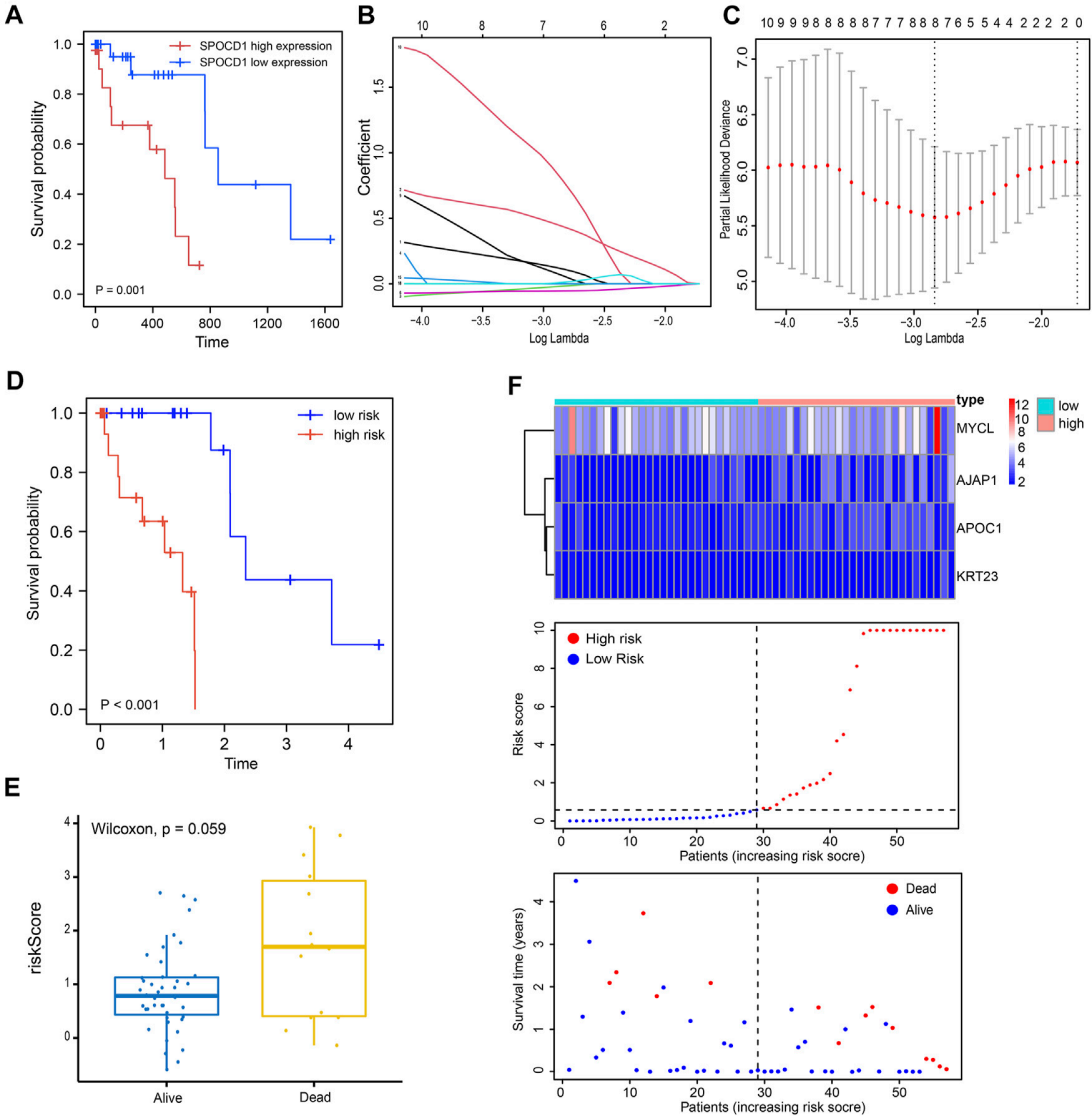
Prognosis correlation analysis of SPOCD1. **(A)** The prognosis of the low SPOCD1 expression group was significantly better than that of the high expression group; **(B,C)** From Lasso regression to multivariate Cox regression: λ and cv maps showed that eight factors were screened; **(D)** The prognosis of patients with the high-risk score was worse than that of patients with low risk; **(E)** The risk score of the death group was significantly higher than that of the survival group; **(F)** Risk heat map and scatter map of independent prognostic genes in multivariate Cox regression. SPOCD1, SPOC Domain Containing 1.

To assess the prognostic value of SPOCD1, the survival information of patients was obtained from the TCGA-ESCC dataset. It was found that the prognosis of the group with high expression of SPOCD1 was significantly worse than that of the group with low expression of SPOCD1 (Figure 10A). To analyze the prognostic genes associated with SPOCD1, we divided the patients into high expression groups and low expression groups. Univariate cox analysis showed that 17 genes satisfied the condition of *p* < 0.1. Afterwards, lasso regression was used to screen again to remove the factors of over-fitting. The final eight genes were identified to meet the conditions. The eight genes were AJAP1, APOC1, CT83, GTSF1, KLRC2, KRT23, MYCL, and STRA6 (Figures 10B,C). Finally, multivariate cox analysis showed that only MYCL, AJAP1, APOC1, and KRT23 were independent prognostic factors. The prognosis of patients with the low-risk score was significantly better than those with the high-risk (Figure 10D). Although the survival group and death group were not statistically significant (*p* > 0.05), the risk score of the death group was significantly higher than that of the survival group (Figure 10E). As a result, we presented the expression and prognostic risk scores of the four genes in the form of a heat map and scatter map (Figure 10F). The result of SPOCD1 related prognostic genes was shown in Table 6.

**TABLE 6:** Univariate Cox regression, lasso regression, and multivariate Cox regression were used to identify SPOCD1 related prognostic genes.

Gene	Univariate cox		Lasso	Multivariate cox	
	HR	*p*-Value	Coef	Coef	*p*-Value
MYO7B	1.22	0.00	—	—	—
MYCL	1.00	0.01	0.00	0.00	0.01
ITGB6	1.00	0.01	—	—	—
APOC1	1.21	0.02	0.44	1.25	0.00
TRPM1	22.86	0.02	—	—	—
SPOCK1	1.07	0.02	—	—	—
GALNT6	1.01	0.03	—	—	—
HK3	1.19	0.03	—	—	—
SLITRK6	1.08	0.03	—	—	—
FGF9	1.18	0.03	—	—	—
STRA6	1.17	0.03	0.01	—	—
KLRC2	1.72	0.04	0.06	1.60	0.11
AJAP1	1.13	0.05	0.11	0.49	0.00
MMP12	1.10	0.06	—	—	—
GTSF1	0.87	0.07	−0.05	—	—
CT83	0.88	0.09	−0.02	−0.33	0.07
KRT23	1.80	0.10	0.78	2.96	0.00

### Validation of the Expression of Target SPOCD1 in ESCC

**FIGURE 11:**
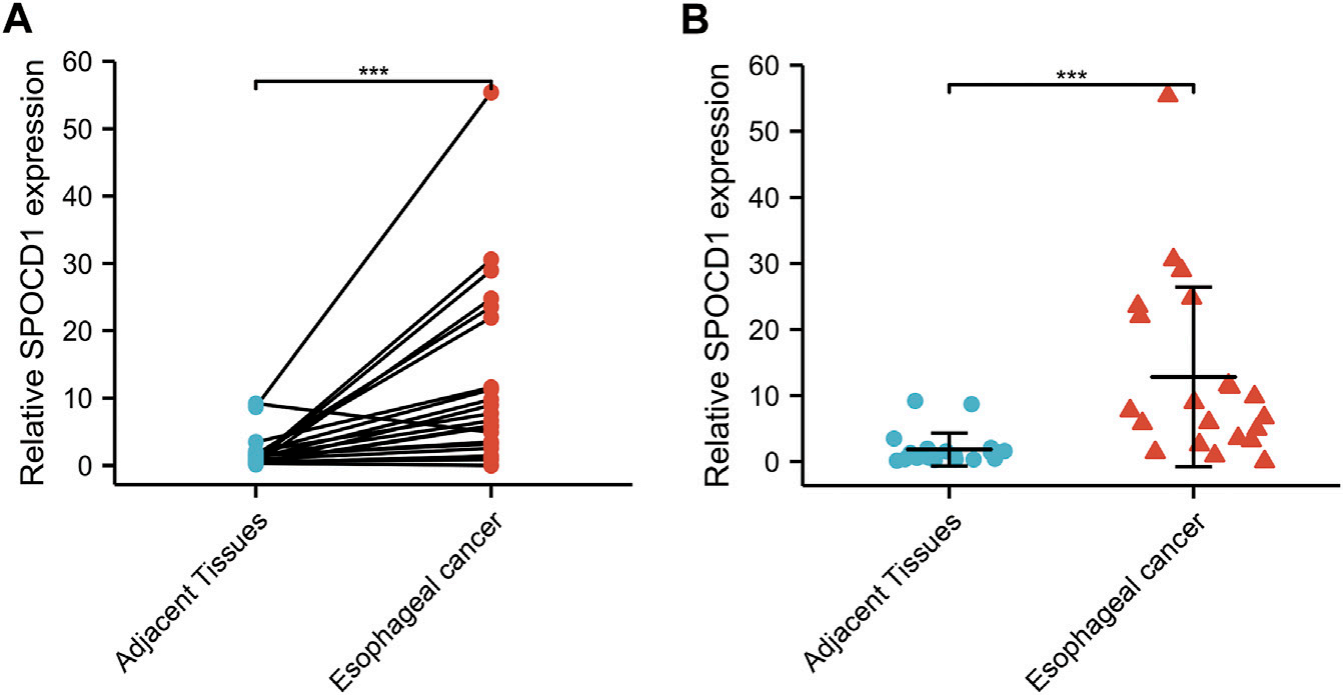
The SPOCD1 expression in ESCC (n = 21), and adjacent normal tissues (n = 21) was evaluated by qRT-PCR. **(A)** The results were analyzed using paired sample *t* test; **(B)** The results were analyzed using Wilcoxon rank sum test. Results expressed as mean ± standard deviation (SD). ***p < 0.001, ***p < 0.001.

Through qRT-PCR, the SPOCD1 expression in ESCC tissues was verified to be significantly higher than that of adjacent tissues using paired sample *t* test and Wilcoxon rank sum test in Figures 11A,B (both *p* < 0.001).

## Discussion

Although the treatment of ESCC is developing rapidly, the prognosis of ESCC patients remains unsatisfied in recent years (Pasquali et al., 2017; Baba et al., 2018). ESCC remains a severe burden on the health system in the world. This is mainly due to the high heterogeneity of ESCC, which leads to significant differences in patients’ responses to treatment (Marshall and Djamgoz, 2018). Even if the patients receive similar treatment and are at the same stage, the clinical results and prognosis are diverse (Navin et al., 2011; Gerlinger et al., 2012). With the continuous development of bioinformatics, a wide range of disease prediction and molecular mechanisms have been gradually acknowledged. Although several studies have been concentrated on this field in the past few years, finding efficient ESCC biomarkers is still an important issue. Therefore, it is critical to identify novel, reliable diagnostic, and targeted therapeutic molecular biomarkers for ESCC, which will improve the effectiveness of diagnosis and treatment, and even increase our understanding of the pathogenesis mechanisms.

In recent years, many studies have revealed that the expression of SPOCD1 was significantly related to the development and occurrence of the tumor, and they may be used as ideal biomarkers of the tumor. SPOCD1 belongs to the TFIIS family that participates in the regulation of development (Kimura et al., 2006). Emerging evidence has indicated that the expression of SPOCD1 is a prognostic factor in several tumors, including gastric cancer, clear cell renal cell carcinoma, ovarian cancer, osteosarcoma, and glioma (Zhu et al., 2017; Liang et al., 2018; Liu et al., 2018; Sakaguchi et al., 2018; Liu et al., 2020). It has been revealed that SPOCD1 can be used to distinguish patients with progressive and non-progressive bladder cancer (van der Heijden et al., 2016). Besides, SPOCD1 promotes the metastasis and proliferation of glioma cells *via* PTX3 (Liu et al., 2018). It also inhibits cell apoptosis and promotes cell proliferation in osteosarcoma through VEGF-A (Liang et al., 2018). Knockout of it decreased gastric cancer invasive activity, cell proliferation, and migration (Zhu et al., 2017). Nevertheless, whether SPOCD1 plays a role in ESCC remains unclear. Herein, we provided a comprehensive analysis of SPOCD1 to illustrate the potential clinical significance and roles in ESCC.

The purpose of this study was to explore the potential role and mechanism of SPOCD1 in the pathogenesis of ESCC through our own sequencing data, ESCC tissue and normal esophageal tissue of TCGA and GTEx dataset. In the present study, 4,435 differential genes were found in the sequencing data of our center, and 4,126 differential genes were found in the TCGA combined with the GTEx data set. After screening, we found that the SPOCD1 gene was differentially expressed in both data sets. Therefore, we selected SPOCD1 as the target gene and used bioinformatics methods to perform the mutation analysis, drug sensitivity analysis, function analysis, pathway analysis, co-expression network analysis, and immune cell infiltration analysis between patients with low and high expression of SPDCD1. Finally, we explored the prognostic significance of SPOCD1.

Our results indicated that the expression of SPOCD1 was significantly increased in ESCC tissues in our independent verification tests and TCGA and GTEx datasets. Interestingly, the same results can be confirmed in other studies. Several studies revealed that SPOCD1 was highly expressed in glioma and gastric cancer (Zhu et al., 2017; Liang et al., 2018). Notably, the analysis of the results showed that the overexpression of SPOCD1 was associated with advanced clinicopathological features and poor outcomes. On the contrary, the expression of SPOCD1 was downregulated in bladder cancer (van der Heijden et al., 2016). The inconsistent expression of SPOCD1 in different tissues may mean that the transcriptional spectrum of different tissue types is different. Given its biological importance in other areas, previous studies and our current study might indicate that SPOCD1 plays a broad regulatory role in human carcinogenesis. These findings supported the suggestion that SPOCD1 might be a promising molecular target for the diagnosis and prognosis of ESCC patients.

It is known to all that SPOCD1 is an oncogene in several tumor types, and plays an essential role in the occurrence and development of tumors. However, its functional way and molecular mechanism in ESCC remain to be elucidated. Therefore, we analyze the function and pathway analysis of SPOCD1. GO and KEGG enrichment analysis of SPOCD1 and its co-expressed genes demonstrated that it may act as an ESCC oncogene by regulating the genes expression in the essential functions and pathways of tumorigenesis, such as glycosaminoglycan binding, Cytokine-cytokine receptor interaction, and Ras signaling pathway. Cytokines can limit the growth of tumor cells through pro-apoptotic or anti-proliferation activity, or by stimulating the activity of immune cells to tumor cells (Berraondo et al., 2019). Ras is involved in the regulation of intracellular signaling pathways, which are involved in fundamental cellular processes such as cell polarity, growth, differentiation, migration, and apoptosis, and eventually lead to cancer (Shields et al., 2000; McKay and Morrison, 2007). Furthermore, we also carried out GSEA to investigate the enriched gene sets and critical pathways. Interestingly, the result showed that the pathway of GSEA enrichment mainly included miR-302c, PRC2 Targets, Multicancer invasiveness signature, adaptive immune response, and adaptive immune response Based on somatic, which was similar to previous findings. Previous studies have indicated that miR-302c expression was significantly correlated with overall survival of locally advanced adenocarcinomas of the gastroesophageal junction (Odenthal et al., 2015). In addition, some literature also demonstrated that PRC2 represses tumor suppressor genes and promotes tumorigenesis (Ha and Kim, 2012; Hu et al., 2012). In the study, multicancer invasiveness signature and adaptive immune response was correlated with the expression of SPOCD1, which revealed that SPOCD1 may play a crucial role in immune response and modulating cancer invasion in ESCC.

To further explore the role of SPOCD1 in tumorigenesis, we also carried out drug sensitivity and immune cell infiltration analysis. The results revealed that SPOCD1 expression was positively correlated with Macrophages M0 and Mast cells activated cells, and negatively correlated with plasma cells and T cells follicular helper cell infiltration. Our functional analysis indicated that the pathway that enriches the most genes was Cytokine-cytokine receptor interaction. Cytokine is the primary regulator of the immune system, and it is also an effective but complex immune mediator. Cytokines can enlarge and activate immune cells and promote the invasion of immune cells to tumors. The manufacture of cytokine-based drugs is a daunting challenge, and an in-depth understanding of the biological role of cytokines is needed to take advantage of their anti-tumor activity while minimizing toxicity.

In the high SPOCD1 expression group, we found certain mutations in TP53, TTN, and MUC16 genes, which were 92, 36, and 18%, respectively. TP53 is a well-known tumor-associated gene for its ability to regulate the malignancy of ESCC cells. Previous studies have shown that TTN mutations are associated with a better response of solid tumors to immune checkpoint inhibitors (Jia et al., 2019). Besides, MUC16 mutation is associated with prognosis and maybe a site affecting tumor prognosis and progression (Yang et al., 2020). In our study, ESCC patients with high SPOCD1 expression indicated poor overall survival, suggesting the expression of SPOCD1 as an underlying factor for the outcome of patients with ESCC. In addition, the mutations that mediate the expression of SPOCD1 possibly have an influence on the development of ESCC. Hence, inhibiting the expression of SPOCD1 might improve the prognosis and therapeutic efficiency of patients with ESCC.

Our multivariate cox analysis showed that only MYCL, AJAP1, APOC1, and KRT23 were independent prognostic factors. MYCL is an oncogene deregulated in human cancers, which supports tumorigenic progression and processes. As therapeutic target, it has been found to be amplified and overexpressed in some malignancies, including gastric cancer and lung cancer (Albihn et al., 2010; Chen et al., 2015; Masso-Valles et al., 2020). Besides, the transmembrane adherens junctions-associated protein-1 (AJAP1) targets the membrane of epithelial cells. Previous research showed that AJAP1 is an independent prognostic factor of squamous cell carcinoma of the esophagus. In ESCC, AJAP1 might serve as a tumor suppressor and that AJAP1 transcription is modulated by hypermethylation (Tanaka et al., 2015). Several researches revealing APOC1 to be a diagnostic and prognostic marker for gastric cancer and colorectal cancer (Yi et al., 2019; Shen et al., 2021). Finally, keratin 23 (KRT23) belongs to the acidic type I keratins (Moll, 1998). KRT23 knockdown decreases proliferation and affects the DNA damage response of colon cancer cells (Birkenkamp-Demtroder et al., 2013). Research have revealed that KRT23 is a subtype-specific prognostic factor for gastric cancer (Min et al., 2017).

Some limitations of our study had to be noted. At present, with the development of high-throughput technology, gene expression profile has become a critical molecular biomarker to identify the phenotype or outcome of ESCC (Zhan et al., 2016). Our study indicated that SPOCD1 might be a momentous biomarker for predicting prognosis in ESCC. Nonetheless, the correlation between the expression of SPOCD1 and the biological mechanisms in ESCC has not been fully clarified. Therefore, further *in vitro* and *in vivo* experiments are needed to validate the biological mechanism of SPOCD1. In addition, more clinical studies are also necessary to identify whether it is an independent prognostic biomarker. Finally, some statistical problems are worth mentioning. 1) A problem of using univariate cox analysis is that this approach ignores the correlation among genes, resulting in inaccurate subset for downstream analysis, especially given that the subset is so small. The network based variable selection methods perform regularized variable selection while incorporating correlations as networks (Wu et al., 2018; Ren et al., 2019), bypassing those disadvantages. 2) Due to the heterogeneity of disease, the heavy-tailed distributions and outliers in the clinical outcomes are widely observed. 3) Our future study should perform variable selection on a much larger scale. The additional gain of using robust network based variable selection is that the identified model is usually more stable and can be easily reproduced. 4) For reproducible research, the gene signature should be reported. The regnet R package can be one of the potential tools for reliable analysis (Wu et al., 2018; Ren et al., 2019). All in all, better statistical methods and more samples are needed to verify our findings in the future.

## Conclusion

In summary, our study indicated that the expression of SPOCD1 was increased in ESCC tissues. The current data support the oncogenic role of SPOCD1 in the occurrence and development of ESCC. Most importantly, SPOCD1 might be an independent prognostic factor for ESCC patients. Our study provided novel evidence into the role of SPOCD1 in the tumorigenesis of ESCC and may promote the development of specific treatments or diagnostics. Further deep investigation and well-designed studies about the exact mechanism of SPOCD1 in ESCC are needed.

## Data Availability

The datasets presented in this study can be found in online repositories. The names of the repository/repositories and accession number(s) can be found below: https://www.ncbi.nlm.nih.gov/geo/query/acc.cgi?acc=GSE194116.

## Supplementary Material

Supplementary Figure S1 Correlation analysis of SPOCD1 expression and mutation. **(A)**. The CNV of the group with high expression of SPOCD1; **(B)**. The CNV of the group with low expression of SPOCD1. SPOCD1, SPOC Domain Containing 1; CNV, copy number variation.

Supplementary Figure S2 Drug sensitivity analysis and drug prediction. **(A–D)**. The differences in IC50 values for drugs prediction with high and low SPOCD1 expression from the GDSC database. SPOCD1, SPOC Domain Containing 1; GDSC, genomics of drug sensitivity in cancer.
