# Supplementary figures and images for: Prognostic Value of SPOCD1 in Esophageal Squamous Cell Carcinoma: A Comprehensive Study Based on Bioinformatics and Validation
Source: Front Genet. 2022 May 11;13:872026. doi: 10.3389/fgene.2022.872026 (PMC9130929; doi:10.3389/fgene.2022.872026)

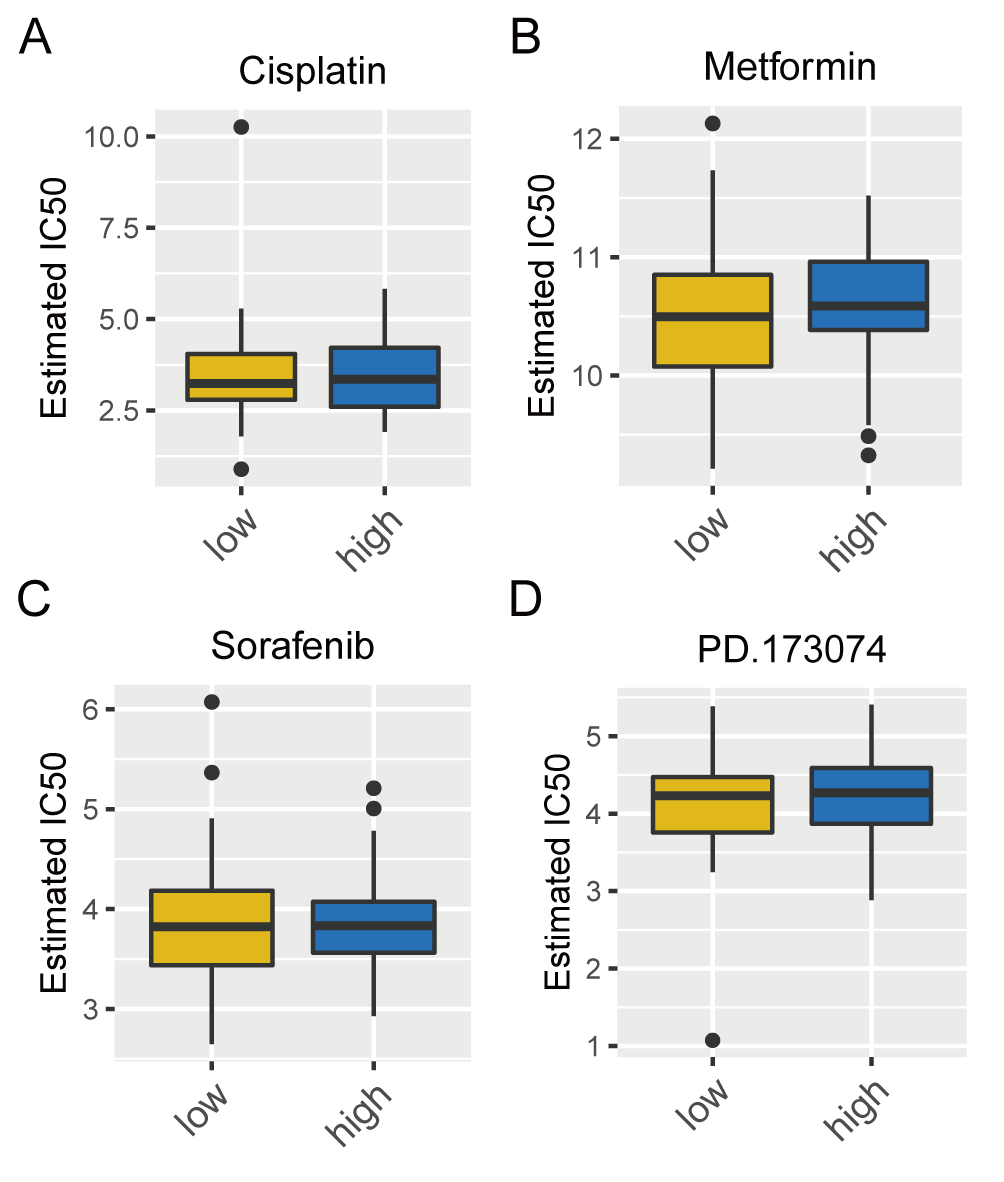

Supplement: Supplementary file 1 [file Image2.TIF]

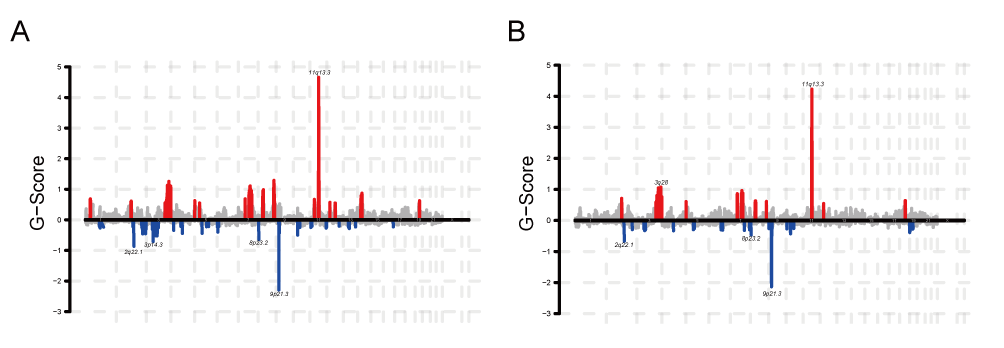

Supplement: Supplementary file 2 [file Image1.TIF]
