# Supplementary material for: Prognostic Value of SPOCD1 in Esophageal Squamous Cell Carcinoma: A Comprehensive Study Based on Bioinformatics and Validation
Source: Front Genet. 2022 May 11;13:872026. doi: 10.3389/fgene.2022.872026 (PMC9130929; doi:10.3389/fgene.2022.872026)
Supplement: Supplementary file 3 [file DataSheet1.docx]

**Supplementary materials**

**Table S1 Clinical data for RNA sequencing of patients with esophageal squamous cell carcinoma in our institution**

| Characteristic | levels | Overall |
| --- | --- | --- |
| N |  | 6 |
| Gender, n (%) | Female | 1(16.7%) |
|  | Male | 5(83.3) |
| Age, n (%) | <=60 | 4(66.7%) |
|  | >60 | 2(33.3%) |
| Smoking, n (%) | Yes | 4(66.7%) |
|  | No | 2(33.3%) |
| Pathologic T stage, n (%) | 2 | 4(66.7%) |
|  | 3 | 2(33.3%) |
| Pathologic N stage, n (%) | 0 | 4(66.7%) |
|  | 2 | 2(33.3%) |
| Pathologic TNM stage, n (%) | I | 3(50.0%) |
|  | II | 1(16.7%) |
|  | III | 2(33.3%) |

**Table S2 Clinical data for qRT-PCR of patients with esophageal squamous cell carcinoma in our institution**

| Characteristic | levels | Overall |
| --- | --- | --- |
| N |  |  |
| Gender, n (%) | Female | 8(38.1%) |
|  | Male | 13(61.9%) |
| Age, n (%) | <=60 | 10(47.6%) |
|  | >60 | 11(52.4%) |
| Smoking, n (%) | Yes | 6(28.6%) |
|  | No | 15(71.4%) |
| Pathologic T stage, n (%) | 2 | 7(33.3%) |
|  | 3 | 13(61.9%) |
|  | 4 | 1(4.8%) |
| Pathologic N stage, n (%) | 0 | 12(57.1%) |
|  | 1 | 3(14.3%) |
|  | 2 | 3(14.3%) |
|  | 3 | 3(14.3%) |
| Pathologic TNM stage, n (%) | I | 2(9.5%) |
|  | II | 11(52.4%) |
|  | III | 7(33.3%) |
|  | IV | 1(4.8%) |
